# The hegemonic EWSR1::ETS oncoprotein overrules core regulatory circuitry principles in Ewing sarcoma

**DOI:** 10.1038/s41698-025-01226-8

**Published:** 2025-12-25

**Authors:** Sandrine Grossetete, Sakina Zaidi, Martin F. Orth, Sarah Morice, Karine Laud, Caroline Louis-Brennetot, Anna Sole Ferre, Virginie Perrin, Florencia Cidre-Aranaz, Virginie Raynal, Marie-Ming Aynaud, Sylvain Baulande, Marco Wachtel, Isabelle Janoueix-Lerosey, Erika Brunet, Thomas G. P. Grünewald, Olivier Delattre, Didier Surdez

**Affiliations:** 1 INSERM U1330, Children’s Oncology Research Unit (CONCERT), PSL Research University, SIREDO Oncology Center, Institut Curie Research Center, Paris, France; 2 Max-Eder Research Group for Pediatric Sarcoma Biology, Institute of Pathology, Faculty of Medicine, LMU Munich, Munich, Germany; 3 Balgrist University Hospital, Faculty of Medicine, University of Zurich (UZH), Zurich, Switzerland; 4 Laboratory of Genome Dynamics in Human Diseases, Equipe Labellisée Ligue 2023, Université Paris Cité, Université Paris Saclay, INSERM UMR 1163, Imagine Institute, Paris, France; 5 University Lyon, Université Claude Bernard Lyon 1, Cancer Research Center of Lyon, INSERM 1052, CNRS 5286, Centre Léon Bérard, 69008 Lyon, France; 6 Hopp Children’s Cancer Center Heidelberg (KiTZ), Heidelberg, Germany; 7 National Center for Tumor Diseases (NCT), NCT Heidelberg, a partnership between DKFZ and Heidelberg University Hospital, Heidelberg, Germany; 8 Division of Translational Pediatric Sarcoma Research, German Cancer Research Center, Heidelberg, Germany; 9 ICGex Next-Generation Sequencing Platform, Institut Curie, PSL University, Paris, France; 10 Lunenfeld-Tanenbaum Research Institute, Mount Sinai Hospital, Toronto, Ontario M5G 1X5 Canada; 11 Institute of Pathology, Heidelberg University Hospital, Heidelberg, Germany

**Keywords:** Cancer, Bone cancer, Cancer genomics, Oncogenes, Paediatric cancer, Sarcoma

## Abstract

Ewing sarcoma is an aggressive bone tumor of adolescence characterized by a hallmark *EWSR1::ETS* fusion oncogene. The resulting chimeric oncoprotein drives tumorigenesis by reshaping transcriptional and epigenetic landscapes. However, how it is transcriptionally regulated and whether additional master transcription factors (MTFs) form a core regulatory circuit (CRC) in Ewing sarcoma remain unclear. Using an extensive panel of Ewing sarcoma cell lines and primary tumors, we mapped super-enhancers and identified enrichment of GGAA microsatellites, confirming their specificity to Ewing sarcoma as compared to other pediatric cancers and normal tissues. Integrating transcriptomic, epigenetic, 3D chromatin conformation, and dependency data, we predicted a set of MTFs potentially forming a CRC. However, functional validation demonstrated that these MTFs neither establish auto-regulatory loops nor confer robust proliferative dependencies typical of CRCs in other pediatric tumors. Instead, EWSR1*::*FLI1 emerged as an “hegemonic” oncoprotein, regulating expression of these MTFs without reciprocal regulation. Knockdown of *EWSR1::FLI1* strongly shifted H3K27ac profiles toward mesenchymal states, whereas silencing individual or combined MTFs did not alter cell growth or *EWSR1::FLI1* expression. These findings highlight the absence of a classical CRC in Ewing sarcoma and emphasize EWSR1*::*FLI1 as the dominant oncoprotein and a major vulnerability in this disease.

## Introduction

Ewing sarcoma is a highly aggressive bone sarcoma that most frequently occurs during adolescence. Genetically, it is characterized by a translocation involving FET (*FUS*, *EWSR1*, *TAF15*) and ETS (E-twenty-six) family members, with *EWSR1*::*FLI1* present in 85% of cases^1,2^. Consequently, a chimeric oncoprotein combining the EWSR1 transcriptional activation and disordered domains with an ETS DNA-binding domain is expressed in all Ewing sarcoma cells. These cells exhibit plastic behaviors, primarily regulated by EWSR1::FLI1 direct and indirect mechanisms^3^. Ewing sarcoma cells proliferate optimally at specific levels or activity of EWSR1::FLI1 but acquire mesenchymal features and eventually die when this oncogene is either silenced (*EWSR1::FLI1* KD) or overexpressed (*EWSR1::FLI1* OE)^3–7^. Fluctuations in EWSR1::FLI1 expression enhance migration and metastasis in preclinical models, although this has yet to be demonstrated in patients. Mechanistically, post-transcriptional/translational modifications, competitive DNA binding, and 3D chromatin alterations can affect EWSR1::FLI1 level or activity^5,6,8–10^. However, the transcriptional regulation of EWSR1::FLI1 remains poorly understood. Beyond its pathognomonic translocation, Ewing sarcoma exhibits few recurrent genetic alterations, primarily involving *STAG2*, *CDKN2A*, *TP53*, and chromosomes 1q, 8, 12, and 16q^11–13^. The pioneer transcription factor (TF) activity of EWSR1::FLI1, which generates neo-enhancers by binding GGAA microsatellites (mSats), plays a pivotal role in tumorigenesis by altering the epigenetic and 3D chromatin landscape^8,14–18^. The high variability in mSat composition among tumors, however, leads to strong intertumoral transcriptional heterogeneity^19,20^ as demonstrated at mSats that regulate *EGR2*, *MYBL2*, *SOX6*, and *PRC1*^21–24^. Despite this variability, Ewing sarcoma shows distinct methylation and transcriptomic profiles relative to other sarcomas or pediatric cancers^18,25,26^.

In normal tissues and various cancers, epigenetic profiling has proven effective in defining cell identity through core regulatory circuitry (CRC) analyses. There, a restricted set of master transcription factors (MTFs), typically co-enriched at super-enhancers (SEs), dominate the control of the gene expression program^27–29^. These MTFs collectively regulate their own expression, forming interconnected auto-regulatory loops. Altering CRCs, for example, through MTFs abrogation, typically alters cellular identity or viability^29^. In several cancers, including pediatric ones, CRCs have been characterized and functionally validated. The depletion of their respective MTFs typically leads to dependency phenotypes. Transcriptional auto-regulatory loops and dependencies are observed as early as 24 and 48 h, respectively, following MTF silencing, and these effects persist over extended periods of time^30–33^. For example, in alveolar rhabdomyosarcoma (aRMS), the fusion oncogene PAX3::FOXO1 (or PAX7::FOXO1) binds SEs together with other MTFs, including MYOD, MYOG, and MYCN, to sustain proliferation, and their depletion leads to altered proliferation and cell death^33,34^. In neuroblastoma (NB), subgroup-specific CRCs have been identified. For instance, the noradrenergic (NorNB) CRC encompasses PHOX2A/B, HAND1/2, and GATA2/3 MTFs, while the mesenchymal (MesNB) CRC is driven, among others, by factors of the AP1 family^30,35^. Single-cell data in these cancers support these findings and have also identified intermediate/transitioning cells, highlighting the strong plasticity of these cancer cell populations^36–38^. In Ewing sarcoma, a CRC including KLF15, TCF4, and NKX2-2 MTFs that cooperate with EWSR1::FLI1 was reported in A-673 and EW-8 cells^39^. Short-term siRNA silencing of individual MTFs decreased cell viability, which was, however, not observed in long-term CRISPR knockout experiments^40^. Given the transcriptional heterogeneity of Ewing sarcoma, studying multiple models is crucial for identifying CRCs^19,41^. Here, we investigated a Ewing sarcoma CRC using a large set of Ewing sarcoma cell lines and tumors, compared to other normal and pediatric cancer samples. Through integrative analyses involving transcriptomics, epigenetics, 3D chromatin conformation, and silencing approaches, we show that the predicted CRC does not point towards Ewing sarcoma vulnerabilities. This contrasts with findings in NB and RMS, where CRC analyses uncovered functional dependencies. Here, we use the term hegemonic (from the Greek *hēgemonikos*, meaning ruling, dominant, or supreme) to describe the overarching influence of the EWSR1::ETS oncoprotein, which overrules the CRC principles in Ewing sarcoma. Altogether, the hegemonic EWSR1::ETS fusion oncoprotein operates upstream of the predicted CRC, represents a key vulnerability, and drives Ewing sarcoma identity.

## Results

### The epigenetic landscape of Ewing sarcoma

**Fig. 1:**
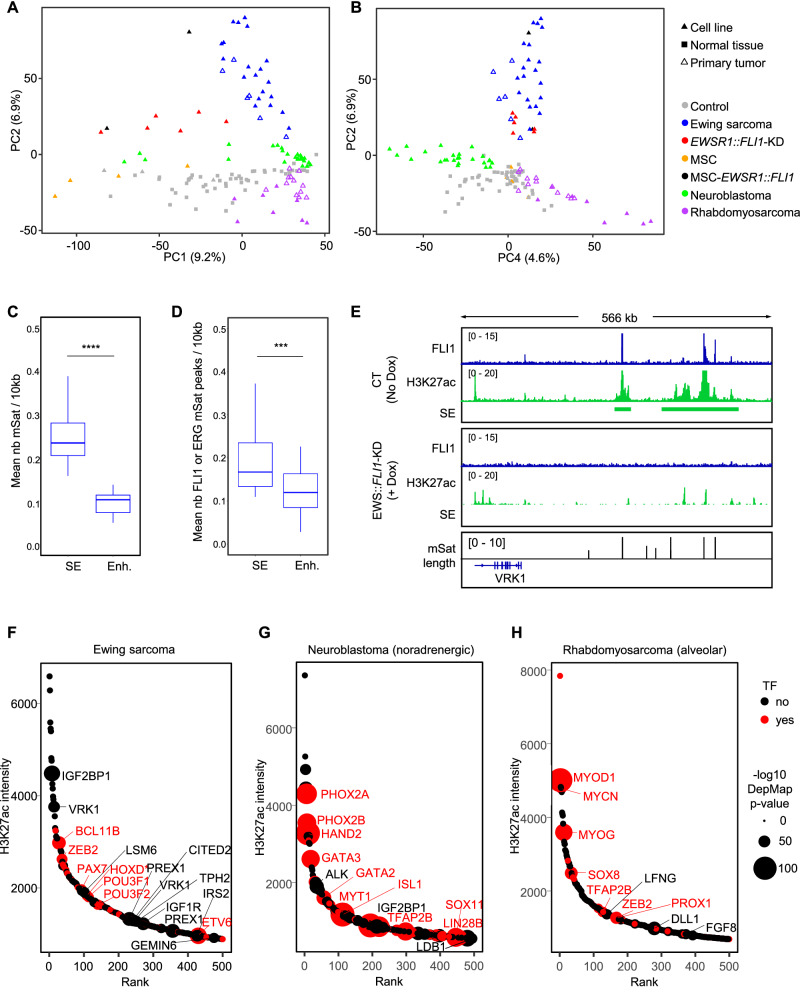
Super-enhancers in Ewing sarcoma. **A**, **B** PCA of normalized intensity in Super-Enhancers for Ewing sarcoma (WT in blue and *EWSR1::FLI1*-KD in red), NB (green), MSC (orange), RMS (purple) and public control data (gray). **C** Boxplot showing the mean of the normalized number of mSats per region per sample (20 Ewing sarcoma cell lines). Only H3K27ac peaks in super-enhancer or enhancer regions were considered. Outliers are not shown. **D** Boxplot of normalized number of mSats ETS peaks per region per sample (20 Ewing sarcoma cell lines). Only H3K27ac and ERG/FLI1 peaks in Super-Enhancer or Enhancer regions were considered. Outliers are not shown. A paired Wilcoxon test was used to compare super-enhancers and enhancers in Ewing sarcoma (**C**, **D**). **E** Example of a super-enhancer specific to Ewing sarcoma with high density of GGAA mSats (*VRK1* locus). IGV was used to represent the track for FLI1 ChIP-Seq (blue), H3K27ac ChIP-Seq (green) and SEs (green bars) for the control (no Dox) and *EWSR1::FLI1*-KD (+Dox) condition in A-673/TR/shEF1 cell line. The black barplot represents the number of consecutive GGAA in the human genome. **F**–**H** Super-enhancer ranked by mean intensity in Ewing cell lines (**F**), norNB cell lines (**G**), and aRMS cell lines and tumors (**H**). Super-enhancers associated with transcription factors are shown in red. The size of the points corresponds to the adjusted *p* value adjusted of genes with Ewing sarcoma (**F**), NB (**G**), and RMS (**H**) dependency based on DepMap 23Q4 data. Only genes with *p* adjust <1^e-5^ for Ewing sarcoma data and RMS, and *p* adjust <1^e-20^ for NB are displayed.

To identify key drivers of Ewing sarcoma identity, we investigated SEs based on H3K27ac ChIP-Seq profiles performed in a panel of 20 Ewing sarcoma cell lines, six primary Ewing sarcoma tumors, two Ewing sarcoma models derived from MSCs (MSC-*EWSR1::FLI1*), and six *EWSR1::FLI1*-silenced Ewing sarcoma cell lines (*EWSR1::FLI1*-KD)^8,19,20,42,43^. Since MSCs have been shown to be one of the putative cells of origin for Ewing sarcoma^43^, SEs from four MSC samples were also included in this analysis. As a comparison cohort, SEs identified in 56 normal tissues/cells and other pediatric cancers, including 25 NB and 20 RMS, were used (Supp. Data 1). Principal component analysis (PCA) of normalized SE intensity distinguished clusters corresponding to normal or tumor cell identities (Fig. 1A, B). PC1 primarily separated MSC and *EWSR1::FLI1*-KD samples from the other entities, while PC2 segregated Ewing sarcoma samples. Gene enrichment analyses of SE-associated genes contributing most to PC1 and PC2 confirmed their mesenchymal and Ewing sarcoma signatures, which correspond to these two axes, respectively (Supp. Data 2). PC1, 2, and 4 also isolated RMS and NorNB samples into distinct groups, while MesNB samples clustered closer to MSC samples (Supp. Fig. 1A, B). Ewing sarcoma cell lines, tumors, and MSC-*EWSR1::FLI1* models formed a separate cluster with broad dispersion, reflecting high intertumoral heterogeneity. Silencing *EWSR1::FLI1* strongly reshaped the H3K27ac-based SE landscape (Fig. 1A and Supp. Fig. 1C). In line with previous transcriptomic studies^41,43^, *EWSR1::FLI1*-KD cells formed an intermediate population, bridging Ewing sarcoma cell lines and MSCs. These results highlight the critical role of the fusion oncogene in defining the epigenetic landscape of Ewing sarcoma (Fig. 1A). Through a comprehensive and agnostic analysis of mSats (all possible repeats of [4-mers]_4x_ and [3-mers]_5x_) in SEs, we confirmed that only GGAA mSats were strongly enriched in Ewing sarcoma. No mSat enrichment was detected in non-Ewing sarcoma samples (Supp. Fig. 2). These findings further support previous data highlighting the unique specificity of GGAA mSats for Ewing sarcoma^3^. The frequency of this mSat was significantly higher in SEs than in enhancers even after size normalization (Fig. 1C). These findings are consistent with previous studies on Ewing sarcoma cell lines, which did not perform size normalization^44,45^. In Ewing sarcoma cell lines, 34% of SEs contained mSats, while mSats were identified in only 3% of enhancers. Furthermore, FLI1- or ERG-ChIP-Seq data from 20 Ewing sarcoma cell lines (Supp. Data 3) showed a significant enrichment of fusion oncoprotein binding at mSats in SEs compared to enhancers (Fig. 1D).

Top-ranked SEs in Ewing sarcoma emphasized previously reported highly expressed or direct EWSR1::ETS targets such as *BCL11B*^46,47^, *CAV1*^48^, *CCND1*^49^, *JAK1*^50^, *NKX2-2*^51,52^*, PRKCB*^53^, or *VRK1*^8^ (Fig. 1E). Additional top hits included *HMCN1*, *DLG2*, and *IGF2BP1*, whose functions in Ewing sarcoma remain unknown or poorly characterized (Fig. 1F and Supp. Data 4). As expected, SE-associated genes in Ewing sarcoma cell lines and in *EWSR1::FLI1*-KD cells were respectively enriched in Ewing sarcoma/proliferation and migratory/mesenchymal signatures (Supp. Fig. 3A, B). To explore the relationship between SEs and their associated gene dependencies, we used the Dependency Map (DepMap) CRISPR/Cas9-KO data (21 days) and selected genes with a negative dependency (Tstat <0) in Ewing sarcoma^40,54^. Among these 291 identified dependency genes, 23 were associated with SEs (8%). This proportion is similar to that reported in aRMS (113 dependent genes, 16 associated with SEs, 14%) or NorNB (756 dependent genes, 69 associated with SEs, 9%) (Supp. Data 5). However, in notable contrast with NorNB and aRMS, where SE-associated TFs were readily identified among their respective top ten DepMap dependencies (*PHOX2A*/*B*, *HAND2*, *GATA3* for NorNB and *MYOD1*, *MYOG* for aRMS) (Fig. 1G, H), in Ewing sarcoma, none of the top ten dependencies were SE-associated TFs, despite a similar representation of TFs across all three entities (Supp. Fig. 3C–E). It should be noted that SEs are typically absent at the *EWSR1*, *FLI1*, and *ERG* loci in Ewing sarcoma.

### Absence of a classical CRC in Ewing sarcoma

**Fig. 2:**
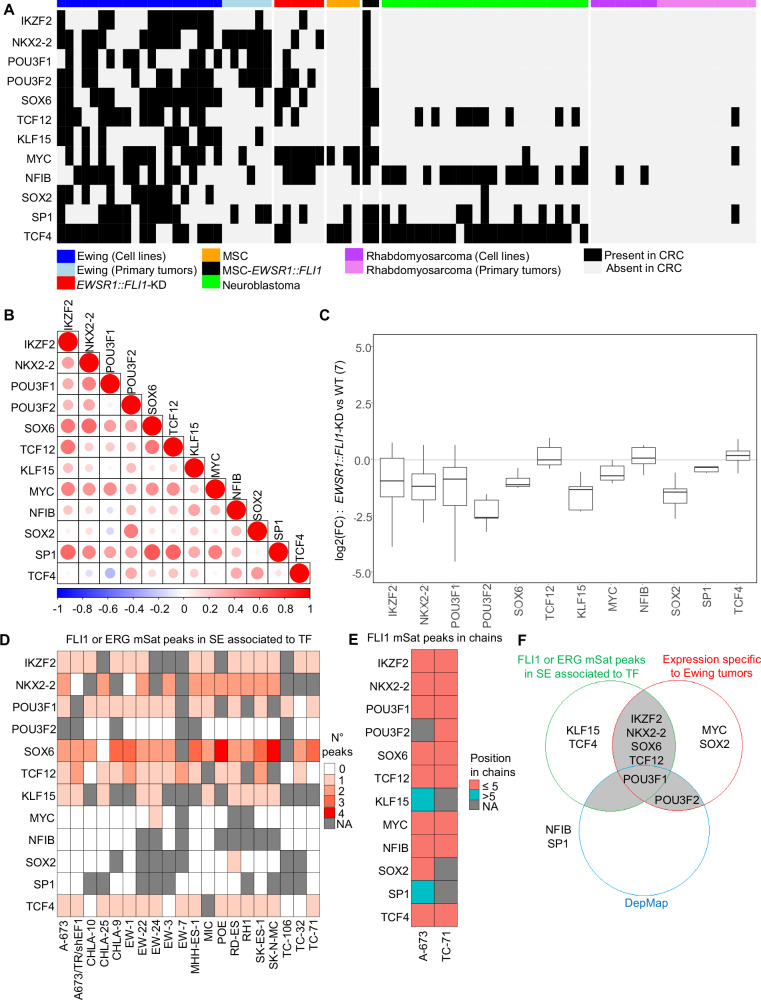
Characterization of CRC candidates in Ewing sarcoma. **A** Heatmap showing the 12 genes predicted to be specific to Ewing sarcoma CRC (≥50% of the Ewing sarcoma cell lines) compared to control data (<30%). Each row represents a gene, and each column a sample. Black and white boxes represent respectively the presence and the absence of the gene in the CRC. The code color at the top is the same as in Fig. 1A. **B** Spearman correlation plot of expression of 132 Ewing sarcoma tumors for the 12 candidate TFs. **C** Boxplot of log2 fold change for the 12 previous candidate genes in each of the seven Ewing cell lines expressing EWSR1::ETS compared to *EWSR1::ETS*-KD oncogene (RNA-Seq data). **D** Heatmap of the number of FLI1/ERG mSat peaks in super-enhancer (SE) associated with the 12 candidate TF. NA values correspond to TFs without an associated SE in the given cell line. **E** Heatmap of position of FLI1 mSat peaks in enhancer-promoter chains in A-673 and TC-71 for the 12 candidate TFs (H3K27ac-HiChIP)^9^. **F** Venn diagram showing the selection of CRC candidates, corresponding to six MTFs that meet at least two of the three criteria (gray area).

A CRC comprises MTFs that typically self-regulate and interact with each other to define cell identity and fate. To identify an Ewing sarcoma-specific CRC, we applied CRC mapper^28^ to our data. We selected MTF candidates identified in at least 50% of Ewing sarcoma cell lines but absent in more than 70% of controls (Supp. Data 6, 7). Twelve MTF candidate genes were identified for the Ewing sarcoma CRC (Fig. 2A). Most of these genes were also identified in Ewing sarcoma tumors and one of the two MSC-*EWSR1::FLI1* CRCs, but were not predicted in MSC, NB, or RMS CRCs. Based on the expression of these 12 MTFs, no sub-clustering within primary tumors could be identified (Fig. 2B). Interestingly, silencing *EWSR1::ETS* in seven Ewing sarcoma cell lines led to a reduction in the expression of nine of these 12 MTFs (Fig. 2C), suggesting their direct regulation by EWSR1::FLI1. To evaluate this, we examined EWSR1::FLI1/ERG-ChIP-Seq and H3K27ac-HiChIP data^9,19^ (Supp. Fig. 4). EWSR1::FLI1/ERG mSat peaks were present in nine SEs associated with these 12 MTFs (Fig. 2D). Cis-regulatory promoter-enhancer (P-E) chains (composed of sequential promoter-enhancer and enhancer-enhancer interactions) incorporating at least one EWSR1::FLI1 mSat-bound element within the first five interactions, were readily identified for ten MTFs in A-673 and/or TC-71 H3K27ac-HiChIP data^9^ (Fig. 2E). This confirms the direct interaction of EWSR1::FLI1 with the promoters of these MTF genes. Some TFs, such as *POU3F2*, did not display EWSR1::FLI1 binding in their closest associated SE. Instead, additional EWSR1::FLI1-bound mSats were connected to these TF promoters through long-range P-E chains (Fig. 2D, E and Supp. Fig. 4). By integrating TFs that^1^ contain mSat peaks within their SEs (Fig. 2D)^2^, exhibit expression specific to Ewing sarcoma across multiple datasets (Supp. Fig. 5A), and^3^ are identified as dependencies in Ewing sarcoma by DepMap (Supp. Data 5), we selected six MTFs (TCF12, SOX6, POU3F1, POU3F2, NKX2-2, and IKZF2) as putative members of the Ewing sarcoma CRC, based on fulfilling at least two out of these three criteria (Fig. 2F).

**Fig. 3:**
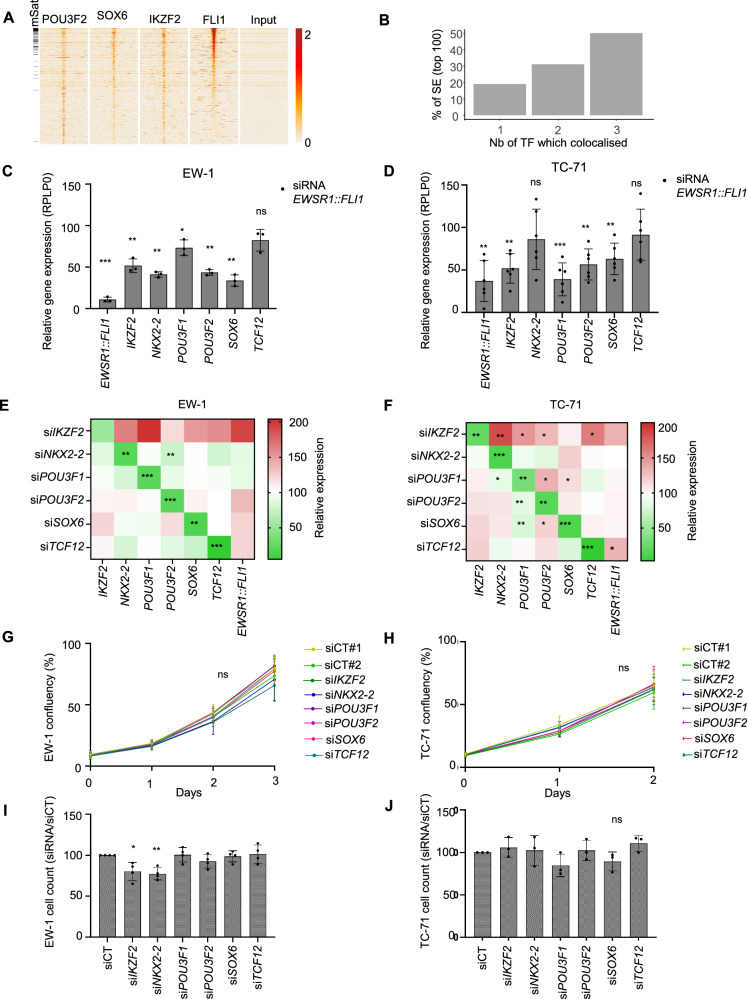
The identified genes are upregulated by EWSR1::FLI1, but they do not form an interconnected co-regulatory circuitry in Ewing sarcoma cells. **A** Heatmap of normalized ChIP-Seq intensity in EW-1 cells for the indicated TFs. 2065 peaks identified in super-enhancers for the three MTF candidates (*POU3F2*, *SOX6*, *IKZF2*) are displayed. The peaks are ranked by their intensity (order: FLI1, mean (POU3F2, SOX6, IKZF2)). Read density is displayed within a 5 kb window around the peak center, and color scale intensities are shown in normalized coverage. The black bar indicates peaks with mSat. Regions with MYC amplification are removed. **B** Barplot showing the distribution of co-bound TF within the top 100 SEs (EW-1 cell line): 1 (only POU3F2, SOX6 or IKZF2), 2 (POU3F2 & SOX6, POU3F2 & IKZF2 or SOX6 & IKZF2), 3 (POU3F2 & SOX6 & IKZF2). Knockdown of *EWSR1*::*FLI1* leads to a decrease in CRC gene expression in EW-1 (**C**) and TC-71 (**D**) Ewing cells. Gene expression analysis by RT-qPCR was performed after transfection with siEF1 and compared to siRNA Control (siCT), and results were normalized using *RPLP0* (48 h post-transfection for EW-1, *n* = 3 and 72 h post-transfection for TC-71, *n* = 6). **E**, **F** Heatmap of RT-qPCR analysis in EW-1 and TC-71, respectively, after transfection with siRNAs and comparing them to siRNA Control (siCT) and normalizing the results using *RPLP0* (48 h post-transfection, *n* = 3). **G**, **H** show proliferation rate using Incucyte technology in EW-1 and TC-71, respectively, after transfecting cells with siRNAs (*n* = 3). **I**, **J** Cell count analysis after siRNAs transfection (3 days post-transfection for EW-1 and 2 days post-transfection for TC-71, *n* = 3). The data were shown as the mean ± SD, *P < 0.05; **P < 0.01; ***P < 0.001 : two-tailed Student’s *t*-test. ns non-significant.

**Fig. 4:**
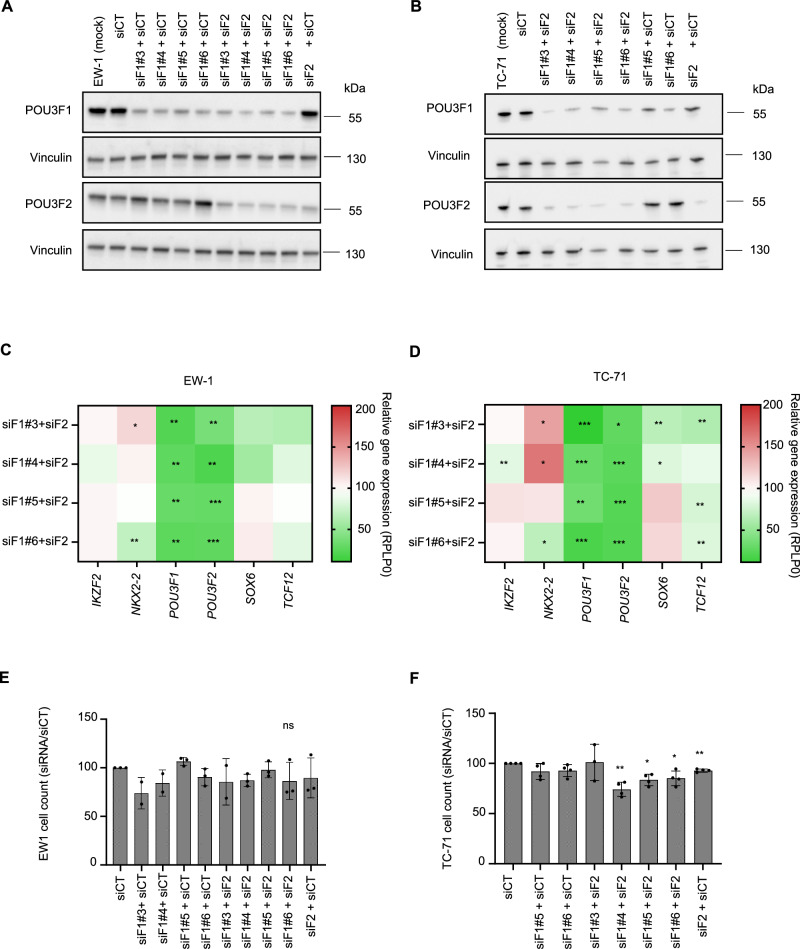
Concomitant silencing of *POU3F1* and *POU3F2* is not sufficient to disrupt Ewing sarcoma putative core regulatory circuitry (CRC). **A**, **B** Western blot analysis for POU3F1 and POU3F2 following siRNA transfection (48 h) in EW-1 and TC-71 cell lines (siF1 = siPOU3F1 and siF2 = siPOU3F2; # indicates different siRNAs). **C**, **D** RT-qPCR-based heatmap in EW-1 and TC-71 cells upon combined *POU3F1/2* silencing compared to siCT conditions (48 h post-transfection, *n* = 2 or 3). **E**, **F** Cell count in EW-1 and TC-71 cells upon single or combined *POU3F1/2* silencing compared to siCT conditions (48 h post-transfection, *n* = 2, 3, or 4).

**Fig. 5:**
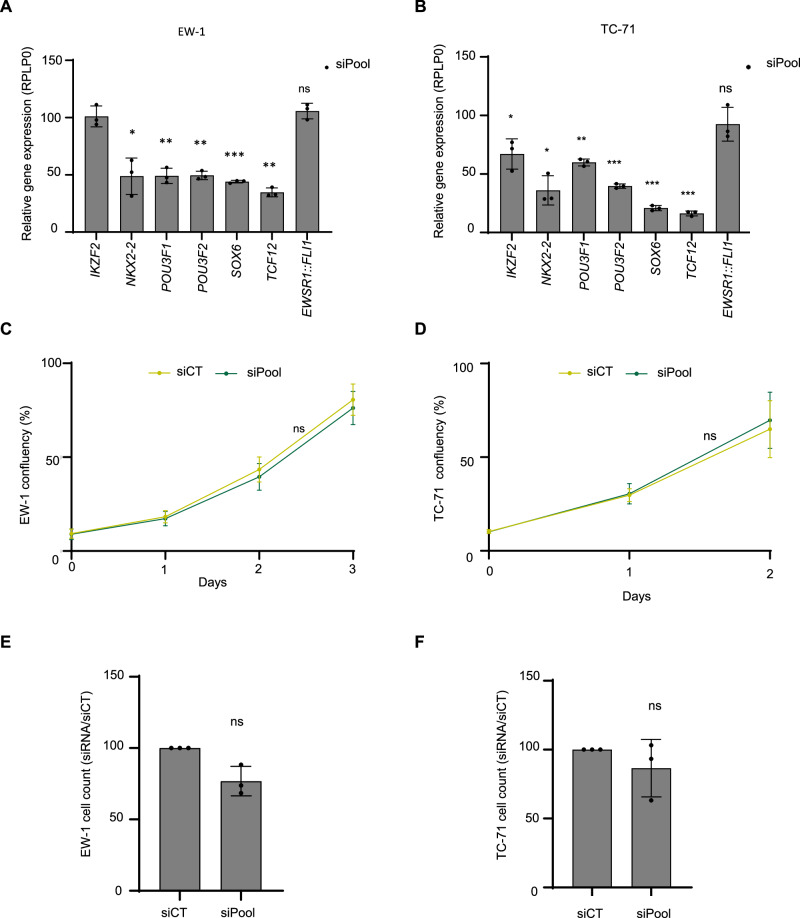
Concomitant silencing of all candidate Master transcription factors (MTFs) is not sufficient to alter the proliferation of Ewing sarcoma cells. **A**, **B** RT-qPCR of MTFs and *EWSR1::FLI1* gene expression in EW-1 (**A**) and TC-71 (**B**) upon pooled silencing of all MTFs (siPool) compared to siRNA control conditions (siCT) (*n* = 3). **C**, **D** Proliferation rates in EW-1 (**C**) and TC-71 (**D**) cells measured using Incucyte technology after siPool compared to siCT (*n* = 3). EW-1 (**E**) and TC-71 (**F**) cell counts upon siPool compared to siCT (*n* = 3). Data were shown as mean ± SD; *P < 0.05; **P < 0.01; ***P < 0.001 (two-tailed Student’s *t*-test); ns not significant. EW-1 data shown 72 h post-transfection, and TC-71 data shown 48 h post-transfection.

To determine if candidate MTFs colocalize with EWSR1::FLI1-bound and SE regions, we performed ChIP-Seq for SOX6, POU3F2, IKZF2, and EWSR1::FLI in EW-1 cell line (Supp. Fig. 5B). Co-localization of MTFs and EWSR1::FLI1 peaks was observed within the SEs associated with these MTFs (Fig. 3A and Supp. Fig. 5C). The three MTFs (POU3F2 & SOX6 & IKZF2) colocalised in 50% of top 100 Ewing sarcoma SEs (Fig. 3B). Using quantitative real-time PCR, we confirmed that silencing of *EWSR1::FLI1* reduced the expression of all, except TCF12, MTFs in EW-1 and TC-71 cell lines (Fig. 3C, D), as observed in RNA-Seq data (Fig. 2C). We then individually silenced these six MTFs in EW-1 and TC-71. However, none of the silencing of candidate MTFs led to a significant decreased expression of the others in both cell lines, hence challenging the interconnected regulatory concept of CRC (Fig. 3E, F). Interestingly, silencing *IKZF2* led to increased expression of the other MTFs, the reverse of positive gene regulation that is expected in CRC models. Moreover, the expression of EWSR1::FLI1 was not modulated upon silencing of these genes (Fig. 3E, F and Supp. Data 8). Despite evidence of co-localization, the lack of co-transcriptional regulation highlights their absence of auto-regulatory function typically expected for MTFs. To evaluate the dependency of EW-1 and TC-71 cell lines on these candidate MTFs, we silenced them and monitored cell proliferation using real-time live imaging microscopy (IncuCyte), but no significant differences were observed compared to the control conditions (Fig. 3G, H). Short-term endpoint cell count experiments confirmed these results (Fig. 3I, J). Silencing of *EWSR1::FLI1* reduced cell proliferation in EW-1 and TC-71 cells, in agreement with previous findings in other Ewing sarcoma cell lines^3,55,56^. Altogether, our data show that, despite partial co-localization, these candidate MTFs do not appear as co-regulatory elements nor acute dependencies in Ewing sarcoma. Nevertheless, these results are restricted to individual MTF considerations. We, therefore, asked if combinatorial silencing of these MTFs would be necessary to impact Ewing sarcoma CRC. Among them, POU3F1 and POU3F2 share 95% homology in their DNA-binding domains. We hypothesized that they could complement each other’s functions. We performed individual and combined silencing of *POU3F1* and *POU3F2* in the EW-1 and TC-71 cell lines and confirmed their specificity and efficiency by RT-qPCR and western blot experiments (Fig. 4A–D). However, combined silencing of *POU3F1* and *POU3F2* did not alter the expression of the other putative MTFs (Fig. 4C, D). Similarly, dual silencing of *POU3F1* and *POU3F2* did not alter proliferation in the EW-1 cell line and had a slight decrease in TC-71 (Fig. 4E, F). Ultimately, we asked whether simultaneous silencing of all MTF candidates could impact *EWSR1::FLI1* level and cell proliferation. A siRNA pool targeting *IKZF2*, *NKX2*-2, *POU3F1*, *POU3F2*, *SOX6*, and *TCF12* was transfected into EW-1 and TC-71 cell lines. Of note, *EWSR1::FLI1* level remained unchanged in the siRNA pool condition (Fig. 5A, B), highlighting the absence of early transcriptional regulatory circuits of these MTFs on the fusion oncogene. Similarly, no impact on proliferation rate was observed under these conditions (Fig. 5C–F). Since a modest but significant increase in the expression of some MTF candidates was observed upon *IKZF2* inhibition in TC-71 (Fig. 3F), we sought to rule out any compensatory mechanism from this gene. To this end, we transfected EW-1 and TC-71 cells with a siRNA pool excluding siIKZF2 and confirmed consistent silencing of the remaining MTFs (Supp. Fig. 6A, B). No or minimal impact on proliferation was observed under these conditions in both cell lines. (Supp. Fig. 6C–F). Altogether, these results show that the predicted CRC does not control the proliferation of Ewing sarcoma cells, at least in the short term.

### Predicted CRC and hegemonic oncogene in Ewing sarcoma

**Fig. 6:**
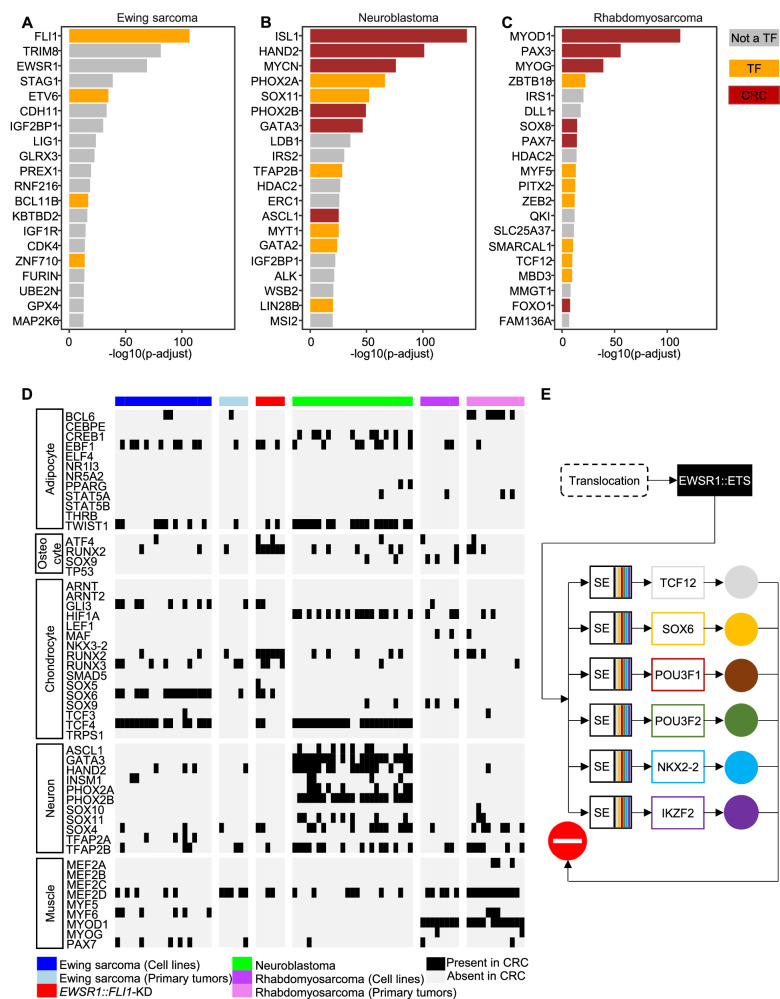
Core regulatory circuitry (CRC) and dependencies in Ewing sarcoma compared to other pediatric cancers and normal tissues. **A**–**C** Bar plots of the top 20 genes with dependencies enriched in Ewing sarcoma (**A**), NB (**B**), and RMS (**C**), using the CRISPR (DepMap 23Q4 public + score, chronos) dataset. The y-axis represents the log10 of the adjusted *p* value from DepMap (dependency in the lineage compared to all other models). CRC genes are shown in brown, TFs in orange, and other genes in gray. **D** Heatmap of TFs involved in different differentiation processes, with only those TFs that have motifs in JASPAR reported. Each column represents a sample, and each row represents a gene. Black boxes indicate TFs that are members of the CRC in the sample. **E** Alternative CRC regulation in Ewing sarcoma.

To investigate whether long-term inhibition of TFs could affect Ewing sarcoma cells, we explored the DepMap database^40,54^. Among our 12 MTF candidates (Fig. 2A), only *POU3F1* and *POU3F2* were reported as dependencies in Ewing sarcoma (among 28 TFs, 291 genes, Supp. Fig. 7A). Notably, these two genes exhibit a moderate effect size as compared to *EWSR1::FLI1*, which ranks as the top Ewing sarcoma dependency in DepMap (Fig. 6A). However, the fusion oncogene is not part of our Ewing sarcoma CRC since SEs were mostly absent around *EWSR1* and *FLI1* loci (Figs. 1F, 6A). The above results are in striking contrast with aRMS and norNB, where MTFs from their CRCs rank among each cancer’s top ten dependencies (Fig. 6B, C) and have been thoroughly validated^30–35,37,40^. Given the well-known heterogeneity of Ewing sarcoma, we investigated whether each tumor might harbor a distinct, individualized CRC. For each cell line, we (1) listed TFs involved in any of the Top 10 predicted CRC and (2) retrieved their TF dependency (gene effect) scores from the DepMap database. However, comparing gene effect scores of CRC TFs with those of non-CRC TFs at the individual cell level revealed no significant enrichment (Supp. Fig. 7B).

Lastly, we investigated if TFs involved in developmental/differentiation pathways of MSCs, the putative cell of origin of Ewing sarcoma, could be enriched in our CRC data. However, TFs controlling adipogenic, osteogenic, or chondrogenic pathways were not enriched in Ewing sarcoma compared to the NB or RMS (Fig. 6D). Altogether, using a broad panel of Ewing sarcoma models, we demonstrate here that the predicted CRC identifies MTFs with predominantly inactive acute transcriptional auto-regulatory and proliferative roles, despite all being direct targets of EWSR1::ETS (Fig. 6E). Instead, we highlight here that the EWSR1::ETS fusion oncoprotein acts as the overarching and unique TF (hegemonic oncoprotein) that regulates a broad panel of heterogeneous target genes and represents the major vulnerability in Ewing sarcoma.

## Discussion

Our findings reveal that the concept of CRC is challenged in Ewing sarcoma and diverges from those observed in other pediatric cancers, such as NB and aRMS. Several distinctions emerge from our analyses that may account for these discrepancies:

Unlike NB or aRMS, Ewing sarcoma harbors SEs that are highly enriched in GGAA microsatellites. We also demonstrated that these mSat-SEs are more enriched in EWSR1::ETS fusion protein binding compared to typical enhancers. These non-canonical elements diverge from evolutionarily conserved regulatory sequences found in developmental gene networks and contribute to a more “neomorphic” mode of oncogenic transcriptional control. By systematically scanning for entity-specific microsatellites in SEs, we confirmed the uniqueness of this feature in Ewing sarcoma. Additionally, some genes associated with SEs appear specific to Ewing sarcoma, as they are defined as Ewing sarcoma dependencies. While genes such as *BCL11B* and *VRK1* are well characterized, others, such as *IGF2BP1* and *LSM6*, remain poorly studied, yet they may offer valuable insights into the biology and vulnerabilities of Ewing sarcoma.

In pediatric tumors, MTFs typically highlight lineage-driven programs. For example, in aRMS, myogenic regulators such as PAX3, MYOD1, and MYOG are co-opted^32^, while in NB, TFs such as HAND2, PHOX2B, and GATA3 govern noradrenergic differentiation and form part of the norNB CRC^30,35^. Notably, these MTFs consistently rank among the top dependencies in their respective cancers, demonstrating strong lineage addiction mechanisms^57^. Interestingly, this observation also emerges in analyses of pediatric circulating tumor DNA (ctDNA). In NB and RMS, fragmentomic studies readily detect lineage-specific MTF footprints^58^. By contrast, previously reported MTFs in Ewing sarcoma^39^ have not proven discriminatory in ctDNA, whereas a broader GGAA-mSat-based enhancer signature robustly distinguishes Ewing sarcoma from other entities^58,59^.

In Ewing sarcoma, the precise cell of origin remains debated. Although bone marrow–derived MSCs can undergo transformation, and *EWSR1::FLI1* silencing reverts 3D chromatin conformation toward an MSC-like architecture^43,51,60^, Ewing sarcoma does not appear to co-opt canonical MSC lineage TFs as principal dependencies. The non-evolutionarily conserved nature of GGAA mSats may explain this divergence, as these elements do not align with standard developmental gene regulatory modules. In contrast, other sarcomas, including RMS, exhibit strong skeletal muscle lineage associations based on phylogeny and gene expression data^61^. Although *EWSR1::FLI1* silencing enables osteogenic differentiation in vitro^41^, raising the possibility of a cryptic MSC differentiation program, our SE analyses (including in *EWSR1::FLI1*-KD cells) did not detect typical MSC lineage-associated MTFs with major functional roles in Ewing sarcoma. These data further support that the fusion oncoprotein’s neomorphic transcriptional activity, rather than a lineage-based CRC, dominates Ewing sarcoma biology.

The expression and enhancer usage of candidate MTFs vary considerably among Ewing sarcoma cell lines, reflecting the extensive intertumoral heterogeneity reported in transcriptomic and methylation studies. Clustering based on MTF or SE occupancy did not reveal biologically meaningful subtypes, underscoring the heterogeneity of Ewing sarcoma epigenomes and highlighting the challenge of defining a universal CRC in this disease. Variability in mSat lengths among Ewing sarcoma cell lines may partly explain this heterogeneity^19,21^. We also hypothesized that cell line-specific vulnerabilities and CRCs might exist in Ewing sarcoma. However, leveraging DepMap data did not reveal such CRCs, although further validation in individual models is needed to fully exclude this hypothesis. Interestingly, the most critical dependencies reported in Ewing sarcoma, aside from the EWSR1::ETS fusion itself, include its E3 ligase TRIM8 and the competing transcription factor ETV6, reinforcing the pivotal role of this fusion oncoprotein^5–7,40^. In NB and aRMS, CRCs have been robustly identified^30,32,35^ and their MTFs are consistently ranked among the strongest dependencies and can be observed as early as 48 h post-silencing in aRMS. However, in Ewing sarcoma, more heterogeneous results have been reported. For instance, silencing of individual MTFs can inhibit proliferation in a subset of Ewing sarcoma lines. Specifically, *SOX6* knockdown impedes proliferation in *SOX6*^high^ lines (RD-ES, TC-32) but not in *SOX6*^low^ lines (A-673), with effects observed 96 hours post-silencing and confirmed in long-term in vivo experiments^23^. Similarly, SOX2 expression is highly heterogenous in Ewing sarcoma tumors, and only 20% express the protein at high levels^62^. *SOX2* silencing reduced proliferation within 48 hours in A-673 and RD-ES but showed little effect in other models^40,54,63^. These results are consistent with a mechanism in which phenotypes correlate with expression levels across diverse cancers rather than being specific to Ewing sarcoma. For example, *SOX2* expression strongly correlates with its knockout dependency across all cancer cell types (Pearson correlation between *SOX2* log₂(TPM + 1) and SOX2 gene effect (Chronos): −0.506, *p* = 8.1 × 10^−73^ ^64^. While this does not rule out functional roles for such TFs in a subset of Ewing sarcoma, it aligns with our findings and the general concept of CRC in defining specific (cancer) entities and vulnerabilities.

A CRC comprising *TCF4*, *NKX2-2*, and *KLF15* was previously reported in Ewing sarcoma. This CRC was inferred from A-673 ChIP-Seq data, refined using CCLE expression databases, and validated in A-673 and EW-8 cells^39^. While we also identified these three MTFs in our putative Ewing sarcoma CRC, silencing *NKX2-2* in TC-71 or EW-1 cells had little to no effect on short-term proliferation and failed to co-regulate the other MTFs. Differences in methods, timing, or cell lines may explain these discrepancies. However, consistent with our findings, long-term knockout of these three MTFs does not impair proliferation in Ewing sarcoma cell lines^40,54^. Our results also agree with previous studies showing that acute *NKX2-2* silencing does not impair proliferation, although clonogenic growth assays and xenograft experiments reveal a strong phenotype^52^. Our findings should, however, not be interpreted to suggest that MTFs have no functional role in Ewing sarcoma. Long-term or context-specific assays, such as clonogenic growth, in vivo tumor formation, or studies under stress conditions, may reveal additional dependencies. Future efforts to map Ewing sarcoma dependencies should consider single-cell or spatially resolved technologies, as our bulk analyses may overlook important nuances. It also remains possible that under specific microenvironmental conditions, during metastasis, or within other cancer hallmarks, certain MTFs become more critical. For instance, POU3F2 may play a role in metastasis, particularly in the context of STAG2 loss^65^. Beyond NKX2-2 and SOX6, whose functions in Ewing sarcoma have already been described and discussed above, the roles of IKZF2, TCF12, and POU3F1 remain to be explored in this cancer.

Our study demonstrates that Ewing sarcoma stands as an exception to the “lineage addiction” paradigm. Rather than engaging with a developmental TF network, the EWSR1::ETS fusion reshapes chromatin by mobilizing a set of GGAA mSats, creating neo-enhancers that drive a neomorphic transcriptional program. Classic auto-regulatory loops involving multiple MTFs do not appear central, as their short-term silencing showed limited impact on Ewing sarcoma cells. Overall, our results suggest that Ewing sarcoma lacks the conventional CRC architecture observed in other pediatric cancers and is instead dominated by a “hegemonic” driver oncoprotein. EWSR1::ETS enforces tumor identity through non-canonical, microsatellite-rich SEs, with minimal reciprocal regulation by MTFs. Despite being still a challenge, our results combined with previous reports support the notion that targeting these dependencies or the microsatellite-based enhancers offer promising therapeutic strategy in Ewing sarcoma.

## Methods

### Cell culture

The Ewing sarcoma TC-71 cell line was obtained from the German Collection of Microorganisms and Cell Cultures (DSMZ). The EW-1 cell line was obtained from the International Agency for Research on Cancer (IARC). TC-71 and EW-1 were cultured at 37 °C in 5% CO_2_ using RPMI (Merk-Sigma, R8758) supplemented with 10% fetal bovine serum (Merk-Sigma, F7524). Cells were routinely tested negative for mycoplasma contamination by qPCR (conducted by Eurofins Genomics). For information on other cell lines, refer to the papers by refs. ^9,19^.

### siRNA

The siRNA experiment was performed as described in our previous paper^9^. Cells were transfected using lipofectamine RNAiMAX (Invitrogen, 13778030) with siCT ON-TARGET plus Non targeting control (Dharmacon, D-001810-01-20) or All-stars negative Control siRNA, (Qiagen, 1027281), siIKZF2 (SMARTpool Dharmacon, L-006946-00-0005), siNKX2-2 (SMARTpool Dharmacon, L-011341-00-0005), siPOU3F1#3 (Qiagen, SI00690221), siPOU3F1#4 (Qiagen, SI00690228), siPOU3F1#5 (Qiagen, SI04166673), siPOU3F1#6 (Qiagen, si04321653), siPOU3F2 (SMARTpool Dharmacon, L-020029-00-0005), siSOX6 (SMARTpool Dharmacon, L-015101-01-0005), siTCF12 (SMARTpool Dharmacon, L-006356-00-0005), siEWSR1-FLI1 fusion type 1 (7/6) for TC-71 or type 2 (7/5) for EW-1 (Qiagen, custom design, see ref. ^9^). For a 24-well plate, siRNA transfection mix was prepared by adding 1 µL of Lipofectamine RNAiMAX to 50 µL of Opti-Mem (Thermo Fisher, ref 31985062) and combining it for 20 min with 50 µl of Opti-Mem and siRNA mix. This mix was then added to 400 µL of the respective cell media. The final concentration of siRNA was 50 nM in the single knockdown experiments and 25 nM of each siRNA in the dual knockdown experiments. For the siRNA pools targeting five or six genes, 25 nM of each siRNA was used, for total concentrations of 125 and 150 nM, respectively. The siRNA controls were transfected at the same total concentrations (125 or 150 nM, respectively). All experiments were conducted without antibiotics and scaled up when necessary. The cells were harvested after 48 or 72 h post-transfection.

### Cell proliferation

EW-1 and TC-71 cell growth was measured using an IncuCyte Zoom (Essen Bioscience). Cells were plated at 25,000 cells/well in a 24-well plate or 50,000 cells/well in a 12-well plate. The cells were imaged every hour using a 4X objective for 2 or 3 days. Cell proliferation curves were generated using the IncuCyte software for confluence analysis (percentage). Cell counts were also performed at the end of the experiment (48 or 72 h) using a Vi-Cell counter (Beckman Coulter) based on cell viability assessed with trypan blue.

### RT-QPCR

RT-qPCR was performed following RNA extraction using RNeasy Plus Mini Kit (Qiagen, ref 74134) and reverse transcription was carried out using the High-Capacity cDNA Reverse Transcription Kit (Applied Biosystems, ref 4368814), according to the supplier’s recommendations. Power SYBR Green PCR Master Mix (Applied Biosystems, ref 4367659) was used for RT-qPCRs, and Oligonucleotides were purchased from MWG Eurofins Genomics (Supp. Data 9). Reactions were run on a CFX384 Touch Real-Time PCR Detection System (Bio-Rad) and analyzed using the Bio-Rad CFX Manager 3.1 software.

### Western blotting

Cells were trypsinized, counted, washed with ice-cold PBS and lysed in Laemmli buffer (50 mM Tris-HCL, 2.5 mM EDTA, 2.55 mM EGTA, 2% SDS, 5% Glycerol, 1% Bromophenol blue, protease inhibitor cocktail tablets and 2 mM DL-Dithiothreitol solution) at 10 million cells/ml as described in our previous paper^9^. Nitrocellulose membranes (BIO-RAD, 1704159) were incubated overnight at 4 °C with rabbit anti-POU3F1 (Abcam, ab259952), rabbit anti-POU3F2 (Cell signaling, 12137), and rabbit anti-vinculin (Abcam, ab129002). Membranes were then incubated for 1 h at room temperature with respective anti-rabbit immunoglobulin G horseradish peroxidase (HRP)-coupled secondary antibody (1:3000, NA934, Cytiva). Proteins were visualized using SuperSignal West Pico Plus (Thermo Scientific, 34580) and a ChemiDoc Imaging System (Bio-Rad).

### ChIP-Seq

Chromatin Immunoprecipitation (ChIP) experiments were performed using the iDeal ChIP-Seq kit for transcription factors and for histones (Diagenode), following the manufacturer’s instructions and as described in our previous study^9^. Briefly, Ewing cell lines were fixed for 10 min with 1% methanol-free formaldehyde (28908, Thermo Scientific). Chromatin was sonicated (Bioruptor, Diagenode) for 20 cycles (30 s on, 30 s off) at the “high” setting to generate DNA fragments with an average size of 150–300 bp. The antibodies used for ChIP were Rabbit polyclonal anti-FLI1 (ab15289, Abcam), rabbit polyclonal anti-IKZF2 Helios (A303-618A, Thermo Fisher), rabbit monoclonal anti-POU3F2/BRN2 (12137, Cell Signaling), rabbit polyclonal anti-SOX6 (ab30455, Abcam), and rabbit polyclonal anti-H3K27ac (ab4729, Abcam). For ChIP sequencing, libraries were generated using the TruSeq ChIP library preparation kit (Illumina) and sequenced on an Illumina HiSeq 2500 or NovaSeq 6000 (single-end, 100 bp).

### ChIP-seq processing

Sequencing data were processed using the nextflow ChIP-Seq pipeline^66^. Briefly, reads were mapped with bwa (version 2.19.0)^67^ to the human genome (version hg19). Duplicates or low-quality reads were removed. Peaks were called with MACS2 (version 2.2.6)^68^, 2008) using the “narrow” option for transcription factors and the “broad” option for the H3K27ac histone mark. The fragment size was estimated from the data using MACS for transcription factors, while a fixed fragment size of 200 bp was used for histones. ChIP-Seq data were normalized according to their respective input DNA samples. The ChIP-Seq signal tracks were generated by macs2 using the bdgcmp option (and –m FE to compute fold enrichment between the ChIP and the control). Files were converted to binary format (BigWig) using bedGraphToBigWig. ETS (FLI1 and ERG) ChIP-Seq peaks were considered as mSat peaks if they contained at least four consecutive GGAA. Super-enhancers were identified by applying the ROSE algorithm to H3K27ac peaks^29^. Core regulatory circuits (CRC) were detected using CRCmapper^28^ with an updated DNA motif database (Jaspar version 2022)^69^. The top 10 CRCs were considered to avoid overly stringent results. Super-enhancers were annotated according to CRCmapper (closest active genes) and compared using BEDTools^70^. Super-enhancers overlapping by at least 50% were merged. For the PCA and SE curve, we used the ROSE signal normalized by the maximum value in each sample. For each group, we averaged the normalized signal and ranked the regions to generate the SE curve. We consider only the top 500 SEs in the paper. For IGV visualization, the tracks were normalized relative to the input. For the heatmap, the coverage was normalized by the total number of mapped reads. Transcription factors were annotated using HumanTFDB (version 3.0)^71^. Dependencies for Neuroblastoma, Rhabdomyosarcoma, and Ewing sarcoma were extracted from DepMap (DepMap, Broad (2023). DepMap 23Q4 Public. Figshare + . Dataset. 10.25452/figshare.plus.24667905.v2). ChIP-Seq tracks were displayed using IGV^72^. For the differentiation factors (Fig. 6D), transcription factors with known motifs were extracted from various papers^34,73–78^. For each TF, we checked whether the predicted de novo motif (best hit) was similar to the expected motif from the JASPAR database^69^. For the motif predictions, we used meme-chip^79^ on the top 5000 peaks (±100 bp around summit positions).

### Expression data analysis

For RNA-Seq from Ewing cell lines, reads were aligned with STAR 2.5.3^80^ to the human genome (GRCh37/hg19). The count matrix generated by STAR used the human gene annotation v19 of GENCODE. Genes with fewer than ten reads were excluded. Data were normalized, and differential analysis was performed using the Wald test with DESEQ2 1.20.0^81^. For the published Affymetrix data of inducible models^19^, the GSE176190 dataset was analyzed using GEO2R. Expression analyses from Ewing tumors, HPA, GTEx and TCGA were processed as described by ref. ^18^.

### Gene dependency analysis

The dataset “CRISPR (DepMap Public 23Q4+Score Chronos)“ was used for gene dependency analyses (DepMap, Broad (2023). DepMap 23Q4 Public. Figshare + . Dataset. 10.25452/figshare.plus.24667905.v2)^40,54^). We downloaded dependencies enriched in Ewing Sarcoma, Neuroblastoma and Rhabdomyosarcoma. In the paper, we considered only genes with a negative T-statistic (dependency higher in the considered tumor type compared to the control dataset).

## Supplementary information

Supplementary information

Supplementary Data

## Data Availability

The H3K27ac ChIP-seq public data used in the paper are indicated in the Supp. Data 1 (extracted from GSE106925, GSE116344, GSE129155, GSE150783, GSE176400, GSE61953, GSE83728, GSE90683, and Encode data). Concerning RNA-seq, public data for A-673/TR/shEF1 (WT vs EWSR1::FLI1-KD, PRJNA521683), A-673 (WT vs EWSR1::FLI1-KD, GSE133228), and MSC vs MSC-EWSR1::FLI1 (GSE150783) were used. The additional ChIP-seq data (H3K27ac, FLI1, POU3F2, SOX6, and IKZF2) and RNA-seq data generated for this study were available on GSE293632.
