# Supplementary material for: The hegemonic EWSR1::ETS oncoprotein overrules core regulatory circuitry principles in Ewing sarcoma
Source: NPJ Precis Oncol. 2025 Dec 25;10:36. doi: 10.1038/s41698-025-01226-8 (PMC12819515; doi:10.1038/s41698-025-01226-8)
Supplement: Supplementary file 1 — Supplementary information [file 41698_2025_1226_MOESM1_ESM.pdf]

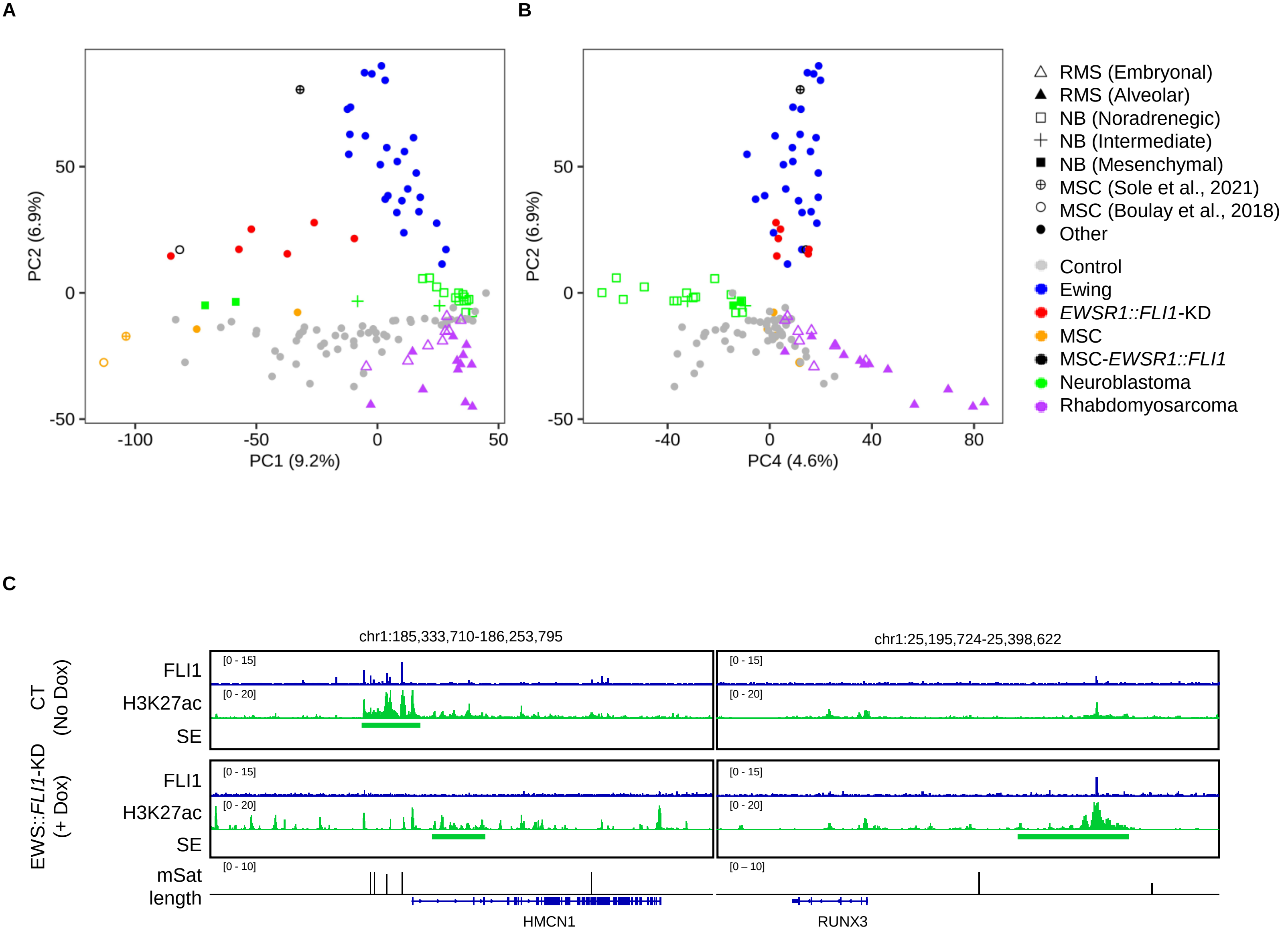

Supplementary Figure 1: Super-Enhancer analyses

(A-B) PCA as in Figure 1, with additional annotations. Shapes specify subgroups in neuroblastoma and rhabdomyosarcoma. MSCs expressing EWSR1::FLI1 are highlighted (42,43). (C) Example of a SE specific to EWSR1::FLI1 basal (left) or EWSR1::FLI1-KD (right) conditions in the A673/TR/shEF1 cell line. ChIP-seq tracks represent H3K27ac (green) and FLI1 (blue) intensity. The black bar plot represents the number of consecutive GGAA sequences in the human genome. Coordinates: chr1:185,585,465-186,264,227 and chr1:25,188,944-25,384,018 (hg19).

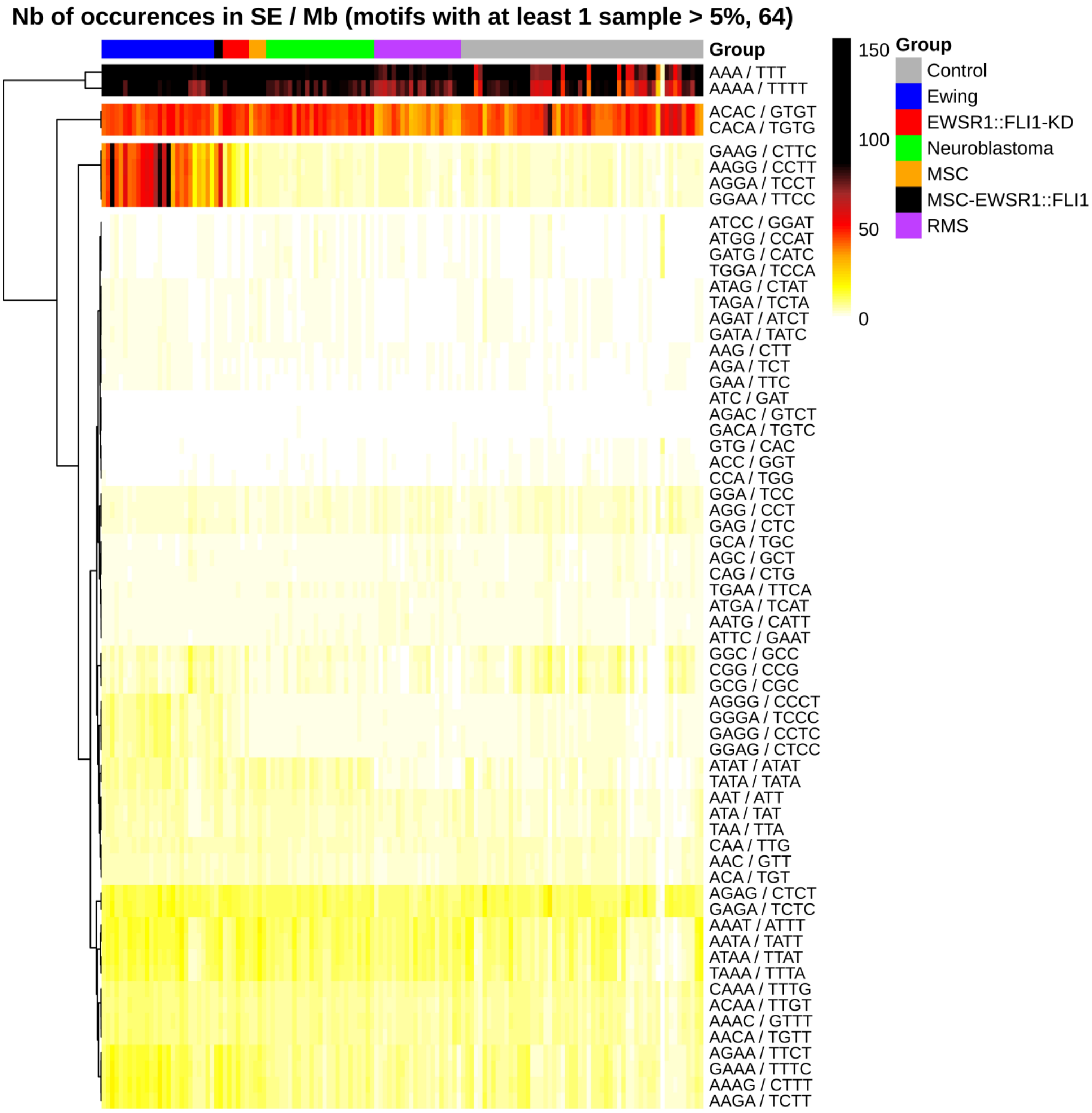

Supplementary Figure 2: Heatmap of motif occurrences in SEs for each sample.

All repeats of [4-mers]4x and [3-mers]5x were scanned across all SEs for each sample. The number of motifs in SEs, normalized to the total length of SEs (Mb), is represented for each sample (color scale from yellow to black). Only motifs found in at least one sample in 5% of the SEs are shown (64 motifs).

A

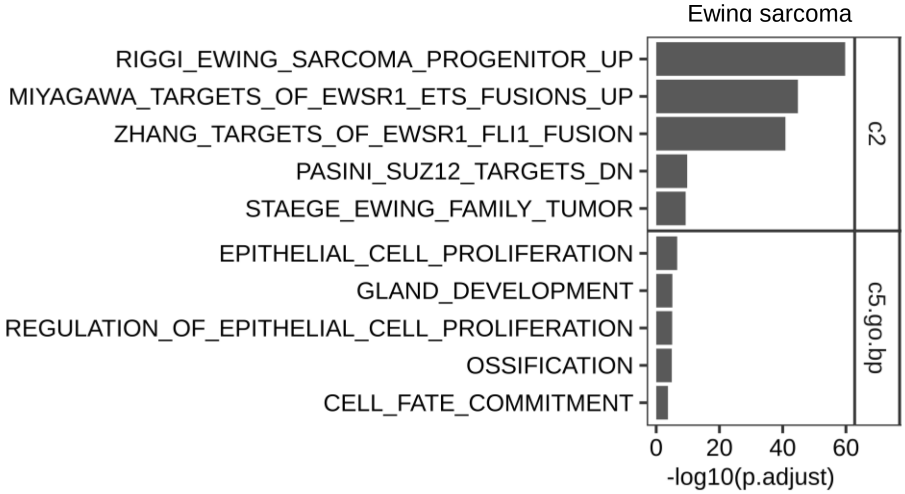

B

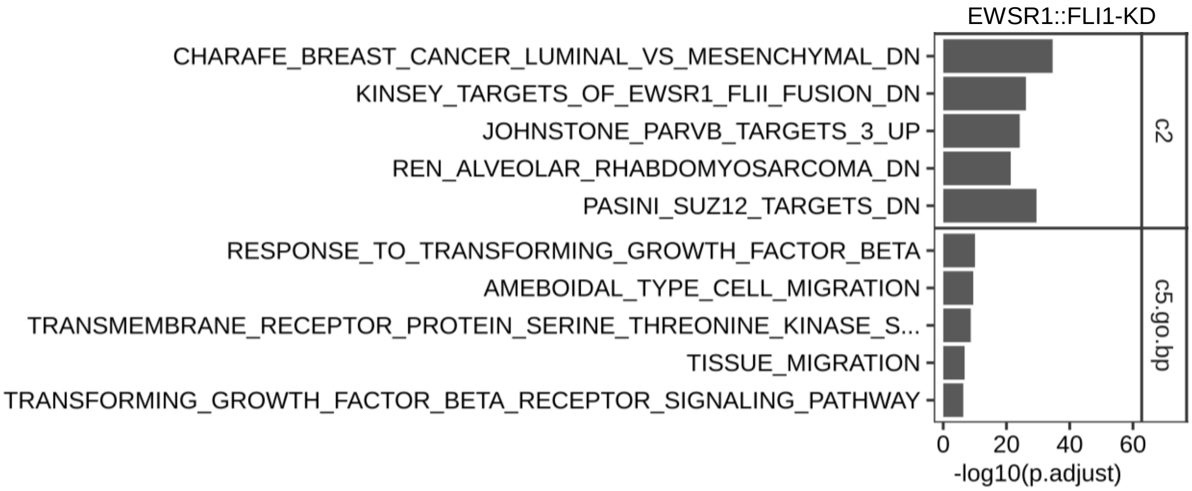

C

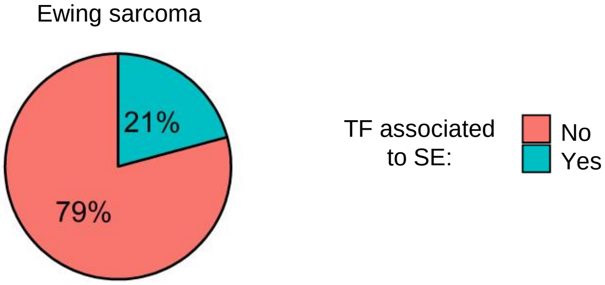

D

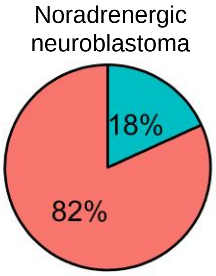

E

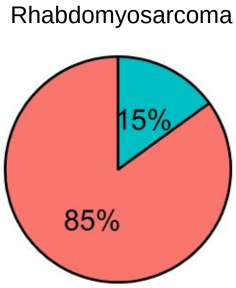

Supplementary Figure 3: Analyses of genes associated to SE
(A-B) Enrichment analysis of genes associated with SEs in Ewing sarcoma (A) and EWSR1::FLI1-KD (B) in the c2 and c5.go.bp databases (source: msigdb_2023.2.Hs). Only the top 5 results are displayed. (C-E) Proportion of TFs in genes associated with SEs in Ewing sarcoma (C), NB (D), and RMS (E).

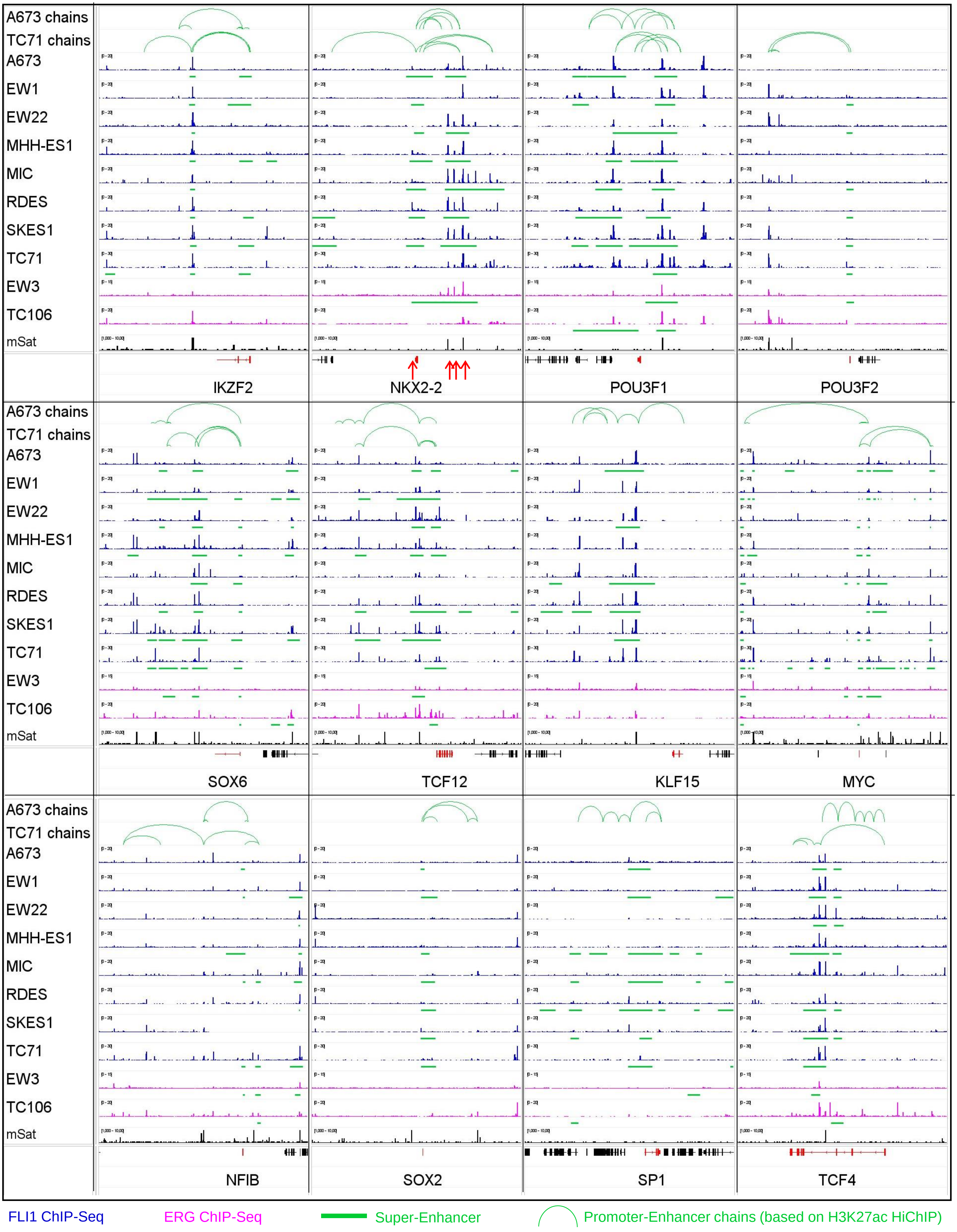
A673 chains
TC71 chains
A673
EW1
EW22
MHH-ES1
MIC
RDES
SKES1
TC71
EW3
TC106
mSat
IKZF2
NKX2-2
POU3F1
POU3F2
SOX6
TCF12
KLF15
MYC
NFIB
SOX2
SP1
TCF4
FLI1 ChIP-Seq
ERG ChIP-Seq
Super-Enhancer
Promoter-Enhancer chains (based on H3K27ac HiChIP)

Supplementary Figure 4: ChIP-Seq and promoter-enhancer interaction of Master transcription factors (MTFs) candidates

Integrative Genomics Viewer (IGV) snapshot around the loci of the 12 MTFs candidates (gene highlighted in red). Promoter-enhancer interaction chains inferred from H3K27ac-HiChIP data (green arcs) in A-673 and TC-71 cell lines (9), FLI1 (blue) and ERG (pink) ChIP-Seq data, and super-enhancers (green lines) for 10 Ewing cell lines. The number of consecutive GGAA sequences in the human genome is shown by the black bar plot. Red arrows for NKX2-2 refer to the 2 ETS and 2 mSat peaks mentioned in discussion.

A

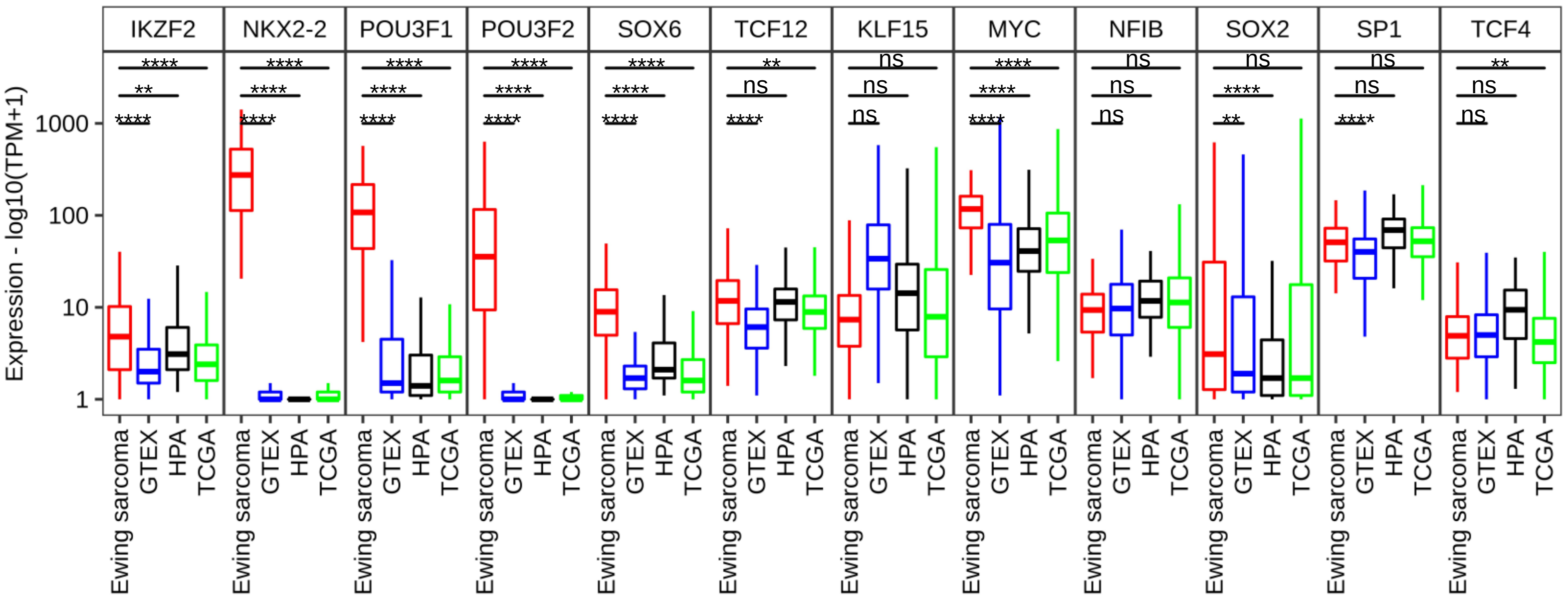

B

ChIP-seq (EW1)	meme-chip results: Motif Found	meme-chip results: Discovery/ Enrichment Program	meme-chip results: E-value	meme-chip results: Known or Similar Motifs	Expected motifs (JASPAR)
FLI1	GAAGGAAGGAAGGAAGGAAGG	MEME	1.2e-881	EWSR1-FLI1 (MA0149.1) IKZF2 (MA2326.1) PRDM9 (MA1723.2)	EWSR1-FLI1 (MA0149.1)
IKZF2	CAGGAA	DREME	5.0e-337	STAT1 (MA0137.4) Stat4 (MA0518.2) IKZF1 (MA1508.2)	IKZF2 (MA2326.1)
SOX6	CATTGT	CentriMo from DREME	1.1e-226	SOX15 (MA1152.2) SOX9 (MA0077.2) Sox3 (MA0514.3)	Sox6 (MA0515.1)
POU3F2	ATGAATAAT	CentriMo from MEME	3.2e-678	POU4F1 (MA0790.2) POU4F3 (MA0791.2) POU4F2 (MA0683.2)	POU3F2 (MA0787.1)

C

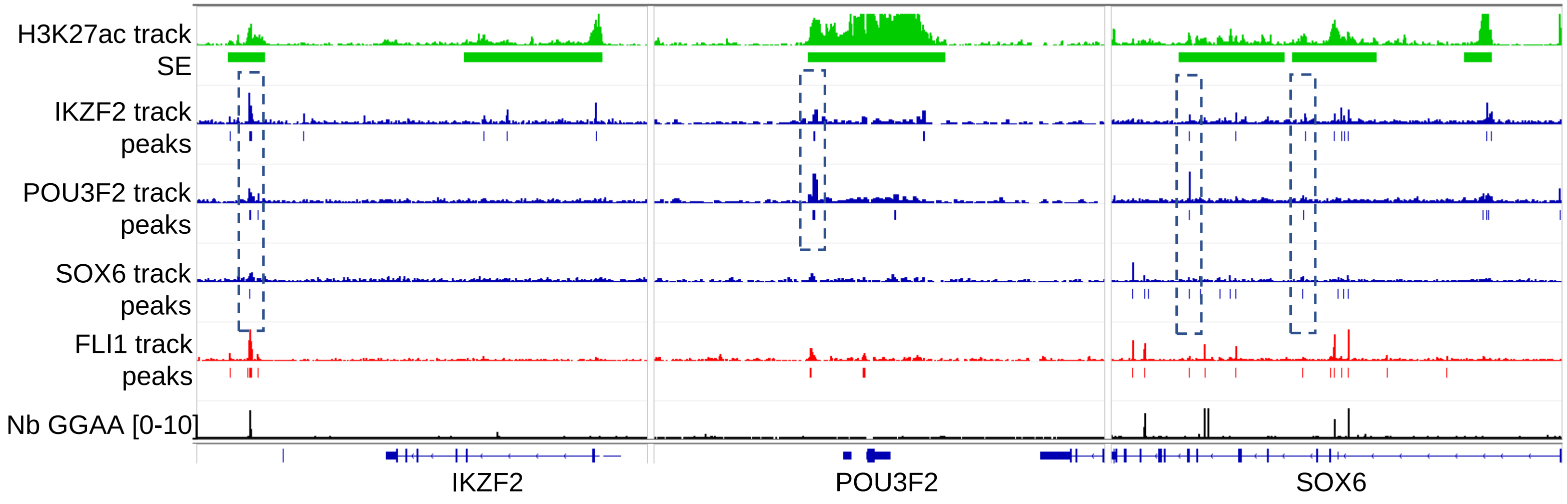

Supplementary Figure 5: Characterization of Master transcription factors (MTFs)
(A) Expression of CRC candidates in 132 Ewing sarcoma tumors (red), TCGA (normal tissue and primary tumors in green), HPA (black), and GTEX (blue). TPM values are computed for each sample. T-test with greater alternative was used to compare expression of Ewing sarcoma tumors versus each of the 3 databases (GTEX, HPA, TCGA)
(B) Motif enrichment results for the top 5000 peaks. For each ChIP-seq in EW-1 cell line, we used meme-chip on the top 5000 peaks (+/- 100 bp around the summit of the peak) and display the top hit. (C) Integrative Genomics Viewer (IGV) snapshot for normalized coverage tracks of H3K27ac marks (green) and IKZF2, POU3F2, SOX6 (blue), and FLI1 (red) ChIP-Seq data in EW-1 Ewing cell line. Peaks called by MACS are indicated. The number of consecutive GGAA sequences in the human genome is shown by the black bar plot. The dashed rectangle represents the colocalization of 2 or 3 tested TF in the S**E.**

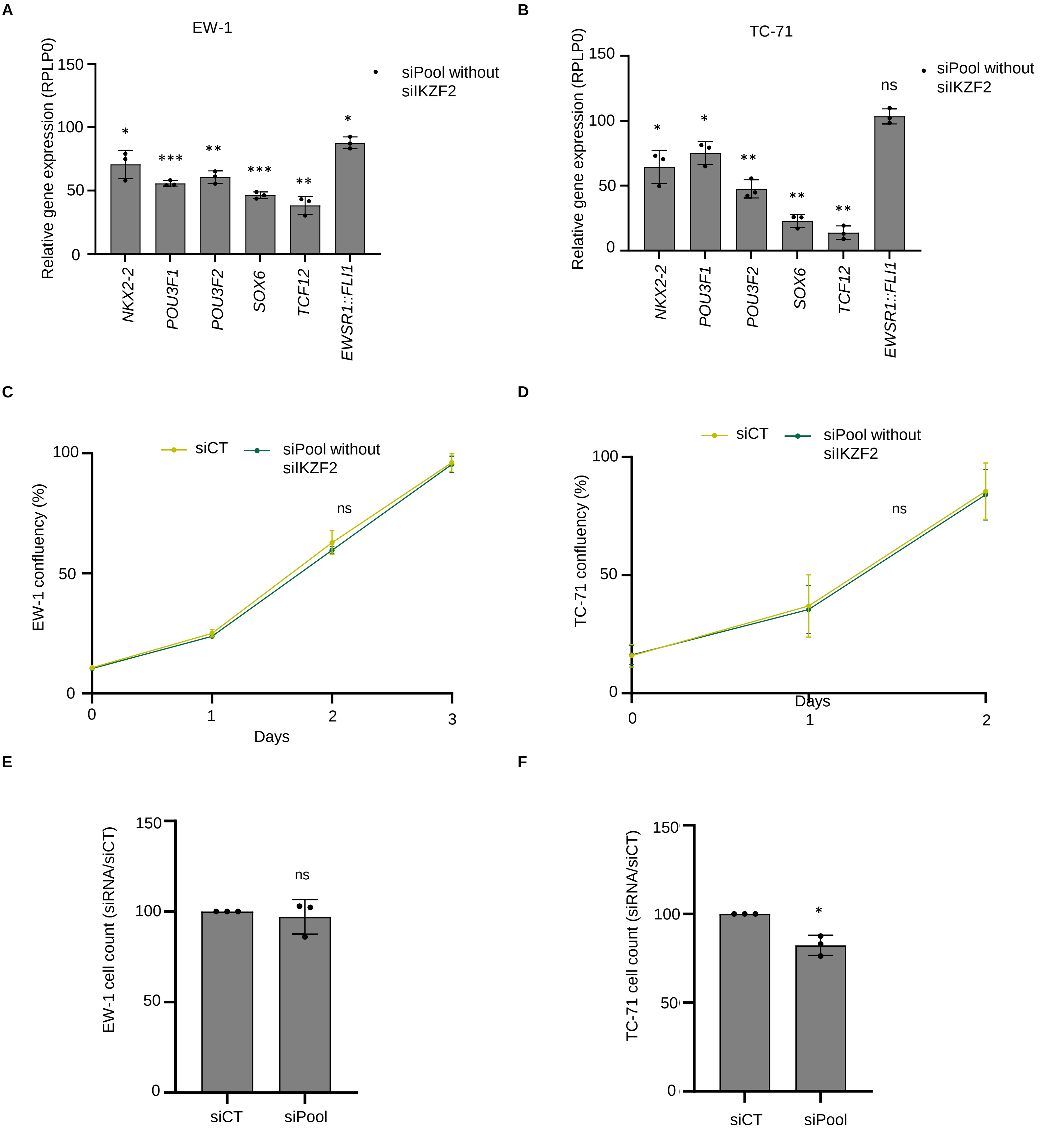

Supplementary Figure 6: Combined silencing of five of the six MTFs (excluding IKZF2) does not impact proliferation of Ewing sarcoma cells

(A-B) RT-qPCR of MTFs and EWSR1::FLI1 gene expression in EW-1 (A) and TC-71 (B) upon pooled silencing of all MTFs except IKZF2 (siPool without IKZF2) compared to siRNA control conditions (siCT) (n=3). (C-D) Proliferation rates in EW-1 (C) and TC-71 (D) cells measured using Incucyte technology after siPool without IKZF2 compared to siCT (n=3). EW-1 (E) and TC-71 (F) cell counts upon siPool without IKZF2 compared to siCT (n=3). Data are shown as mean ± SD; *P < 0.05; **P < 0.01; ***P < 0.001 (two-tailed Student's t-test); ns (not significant). EW-1 data shown 72h post-transfection and TC-71 data shown 48h post-transfection.

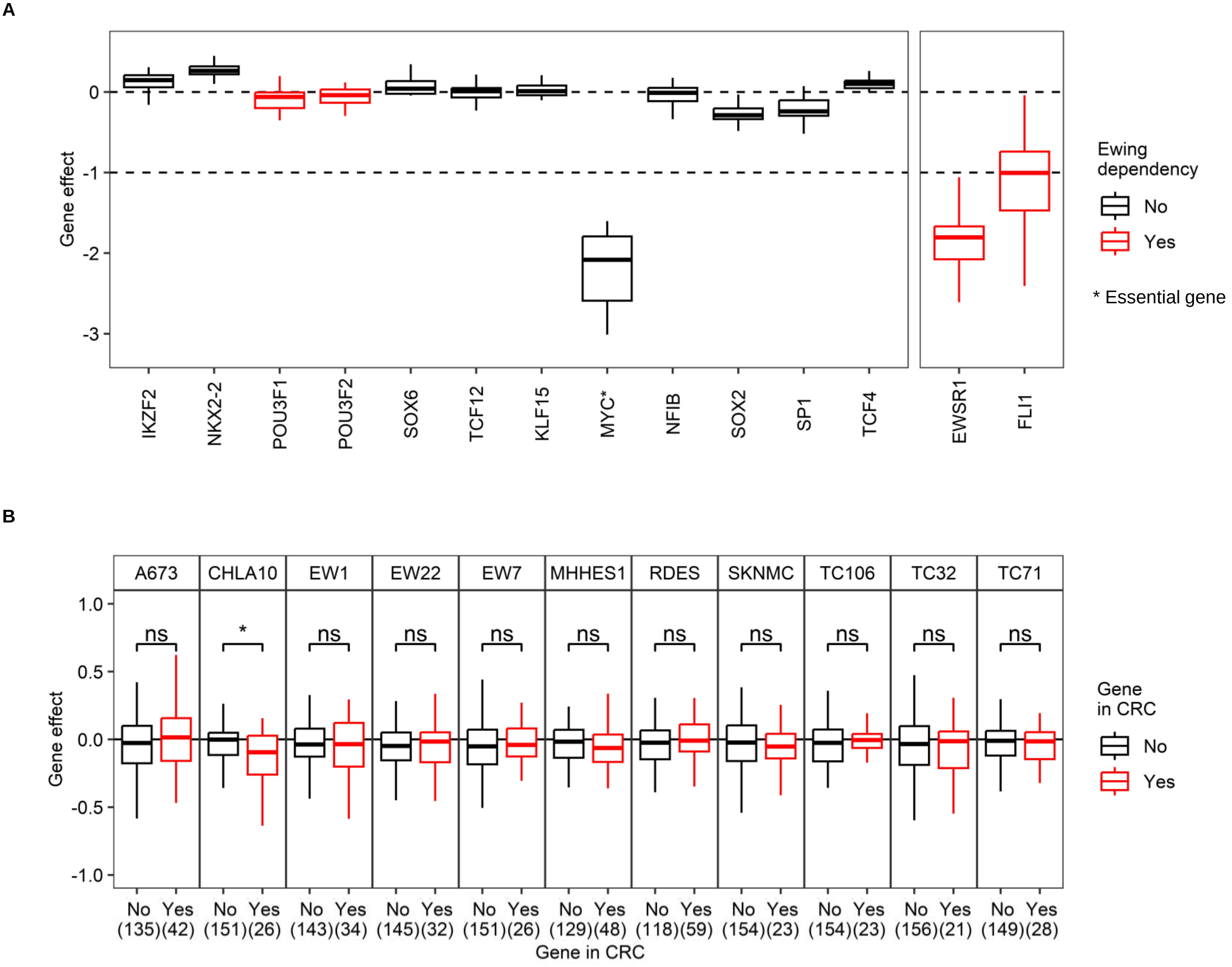

Supplementary Figure 7: Exploration of CRC with DepMap data

(A) Chronos Gene effect in 23 Ewing cell lines for the 12 CRC candidates (from DepMap database). Genes with significant dependency in Ewing sarcoma are indicated in red. The dashed lines correspond to a score equivalent to not essential genes (0) and essential genes (-1). (B) Comparison of gene effects between predicted CRC genes (red) and others (black). Only the 11 Ewing cell lines common between DepMap and our H3K27ac ChIP-Seq data were considered. A Wilcoxon test was performed to compare the gene effects of CRC-involved genes versus others.

Supplementary Data Legend

- **Supplementary Data 1 : Samples used in the study (PCA & CRC)**

- **Supplementary Data 2 : Genes enrichment for genes associated to SE contributing the most to PC1, PC2, PC3, PC4**

- **Supplementary Data 3 : Summary of FLI1 or ERG ChIP-seq for each Ewing sarcoma cell line**

- **Supplementary Data 4 : Super-Enhancer description for each group**

- **Supplementary Data 5 : Super-Enhancer description for each group, one line per gene with annotation (TF, DepMap)**

- **Supplementary Data 6 : Summary of CRC predicted for each group**

Table with percentage of samples with TF in these CRC (top10 of CRC mapper). The number of samples per group is indicated in parentheses (first line).

- **Supplementary Data 7 : CRC predicted for each sample**

List of TFs predicted in its CRC foreach sample (top 10 of CRC mapper)

- **Supplementary Data 8 : RT-qPCR data for Figure 3 E and F**

- **Supplementary Data 9 : List of oligonucleotides used in the paper**

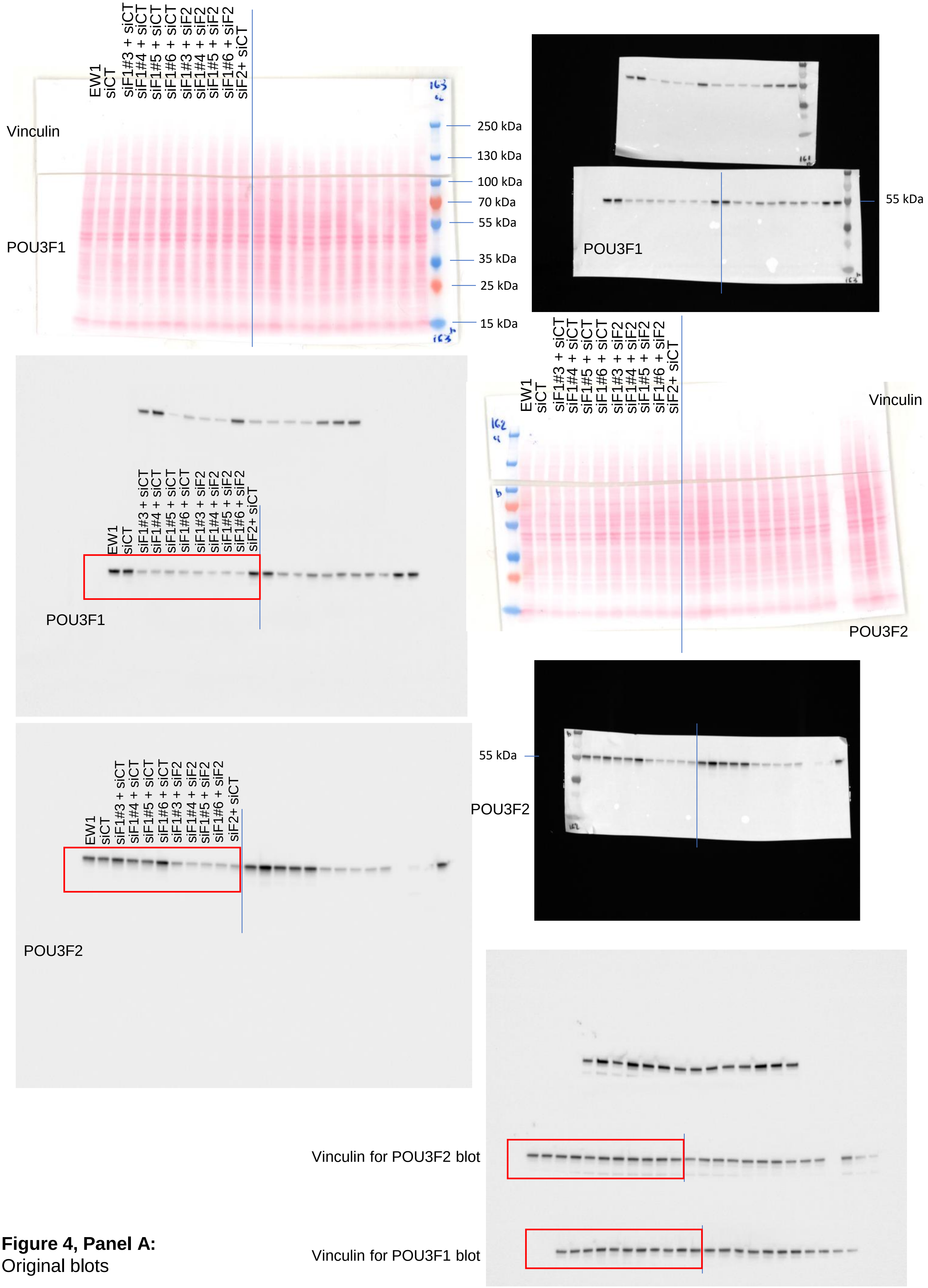

Figure 4, Panel A:
Original blots

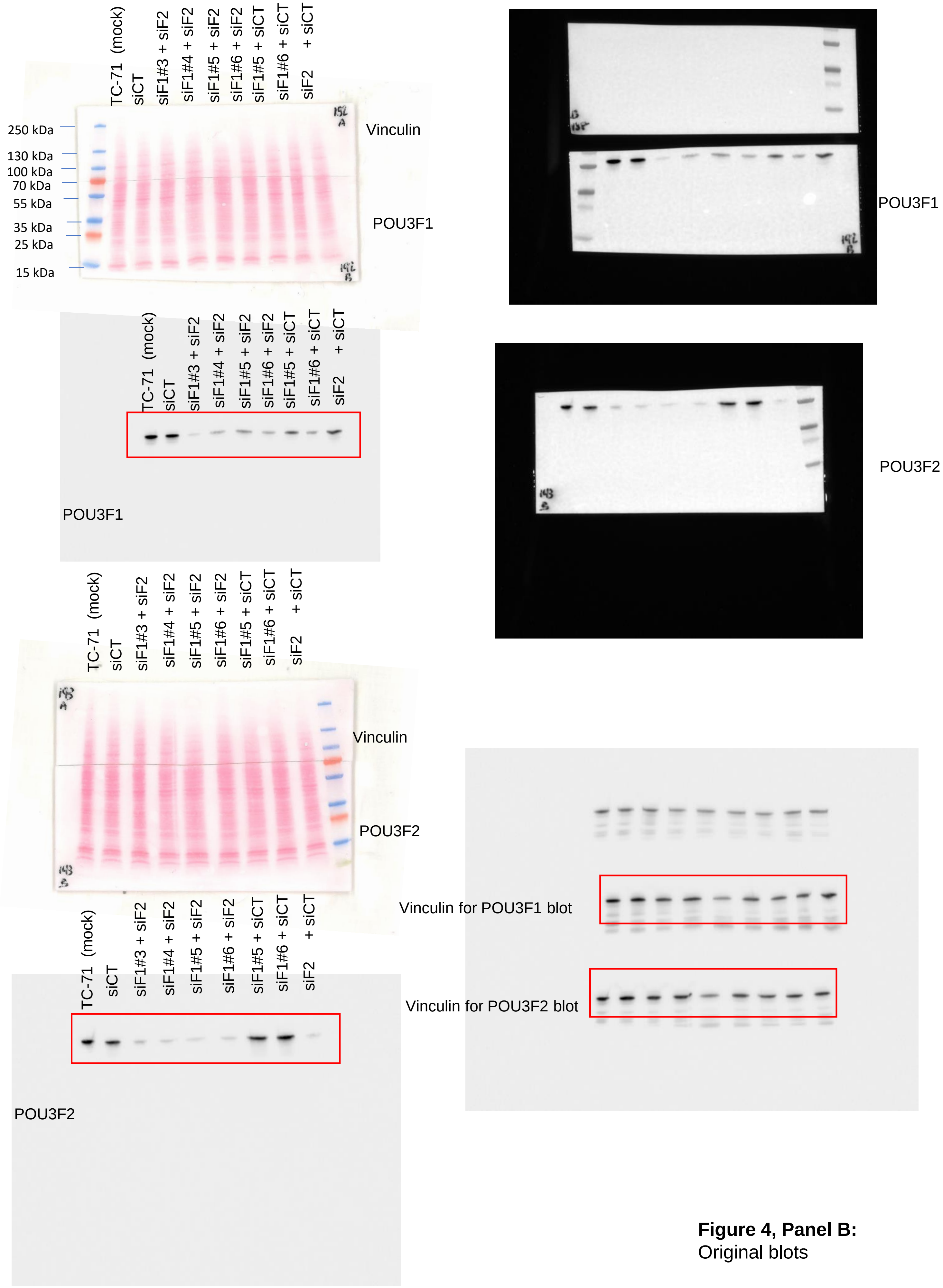

Figure 4, Panel B:
Original blots
